# Anti-Proliferative and Pro-Apoptotic Activities of Synthesized 3,4,5 Tri-Methoxy Ciprofloxacin Chalcone Hybrid, through p53 Up-Regulation in HepG2 and MCF7 Cell Lines

**DOI:** 10.31557/APJCP.2021.22.10.3393

**Published:** 2021-10

**Authors:** Marwa A Eisa, Moustafa Fathy, Gamal El-Din A A Abu-Rahma, Mohamed Abdel-Aziz, Maiiada Hassan Nazmy

**Affiliations:** 1 *Biochemistry Department, Faculty of Pharmacy, Minia University, Minia 61519, Egypt. *; 2 *Medicinal Chemistry Department, Faculty of Pharmacy, Minia University, Minia 61519, Egypt. *

**Keywords:** Ciprofloxacin, apoptosis, P53, Cox-2

## Abstract

**Background:**

Cancer is a significant health problem around the world and one of the leading causes of human death. The need for novel, selective and non-toxic anti-cancer agents is still urging. Aim of the work: to investigate the anti-proliferative and pro-apoptotic effects of the synthesized ciprofloxacin 3,4,5 tri-methoxy chalcone hybrid (CCH) on the HepG2 hepatocellular carcinoma and MCF7 breast carcinoma cell lines.

**Materials and Methods:**

HepG2 and MCF7cell lines were treated with CCH. Cell viability and cell cycle analysis were performed. Protein and mRNA expression levels of P53, COX-2 and TNF-α were analyzed by western blotting and RT-PCR respectively.

**Results:**

CCH caused concentration and time-dependent reduction in the viability of human HepG2 and MCF7 cells, pre-G1 apoptosis and cell cycle arrest at G2/M stage, significantly higher P53 and TNF-α mRNA and protein expression levels but significantly lower COX2 mRNA and protein expression levels.

**Conclusion:**

CCH showed obvious anti-proliferative and apoptosis-inducing activities in both cell lines.

## Introduction

Being one of the most urging health problems worldwide, Cancer is still one of the most difficult medical challenges (Abd El-Baky et al., 2020; Avendan and Menéndez, 2008). Early cancer detection and developing new anticancer drugs remains a critical and challenging goal in medicinal chemistry and thus creating novel effective anticancer drugs is a vital cancer treatment strategy (Abdel-Hamid et al., 2013; Abdellatef et al., 2021). 

Quinolones are known for their antibacterial and antitumor activities through adjusting the normal functions of bacterial gyrase, and are found to be a topoisomerase II inhibitor in human cells (Paul et al., 2007). Ciprofloxacin, a commonly used broad spectrum fluoroquinolone antibiotic, has shown anticancer action in several cancer cell lines (El-Rayes et al., 2002; Herold et al., 2002a). It was shown to induce cell cycle arrest, break DNA doublestrands, and trigger apoptosis of cancer cells (Smart et al., 2008). 

There are many causes indicating that ciprofloxacin might be used as an adjuvant therapy in some cancers (Kloskowski et al., 2011). Aranha et al. noticed that ciprofloxacin can inhibit growth of human hepatocellular carcinoma cell line HepG2 at high doses (Aranha et al., 2000). For getting ciprofloxacin anticancer action, it has to be administered at a much higher concentration than those used for the treatment of bacterial diseases (Smart et al., 2008). At high concentrations (usually 200-300 μg/ml), ciprofloxacin can cause apoptosis of bladder carcinoma cells and may lead to the arrest of the cell cycle at the S/G2 stage (Aranha et al., 2000). 

Major attention has recently been given to the change from antibacterial fluoroquinolones (ABFQs) to antitumor fluoroquinolones (ATFQs), based on their mechanistic similarity and sequence homologies of their target topoisomerase (Mugnaini et al., 2009). Structural modification of clinical ABFQS, especially in the heterocyclic ring, such as piperazine at the 7 position of the quinolone scaffold, has resulted in many ATFQs (Chang et al., 2009). Several studies have suggested that the introduction of a substitution for N-4-piperazinyl moiety of quinolones has altered the physiochemical properties of quinolones, which may influence the cytotoxicity of these compounds and the selectivity of these derivatives to topoisomerase I and II (Yogeeswari et al., 2005). Structure- activity relationship studies revealed that substituting carboxylic acid group with hydroxamic acid, inhibit cell proliferation of lung and colon cancer (Alaaeldin et al., 2021; Alaaeldin et al., 2020). Other studies have documented the synthesis of certain C-7-piprazinyl-ciprofloxacin-chalcone hybrids to enhance the physiochemical properties of ciprofloxacin and/or the synergistic effect by combining ciprofloxacin and chalcone in one structure. The compound was tested for in vitro anticancer activity using cancer cell lines (Abdel-Aziz et al., 2013; Moustafa Fathy et al., 2020).

The cancer-related inflammation is one of the most important mechanisms for carcinogenesis (Fathy and Nikaido, 2013, 2018; Landskron et al., 2014). TNF-α is a protein which weighs 17-kDa, and consists of 157 amino acids. In humans, the gene is located on chromosome 6 (van Horssen et al., 2006). TNF-α action is mediated through two cell surface receptors, TNF-R1 and TNF-R2 that vary in their signaling activity. TNF-R1 is usually proapoptotic while TNF-R2 has an anti-apoptotic action (Cabal-Hierro and Lazo, 2012; Soares et al., 1998). The human COX 2 gene is placed on chromosome 1q25.2 q25.3 and aids in the occurrence and development of tumors by encouraging cell prolif¬eration, stopping cell apoptosis, inducing angiogenesis and suppressing immune reactions (Yu et al., 2016; Chen et al., 2015). The best-known tumor suppressor gene P53, it is located on chromosome 17p13.1 (Morán et al., 2010). In reaction to DNA damage, this gene codes for P53 protein which induces cell-cycle arrest in the G1 phase and promotes DNA repair genes. If a cell with damaged DNA cannot be repaired, the cell is guided by p53 to experience apoptosis (Lukin et al., 2015; Choi et al., 2005).

Consequently, we investigated the anti-proliferative and apoptotic inducing effects of CCH on HepG2 hepatocellular carcinoma and MCF7 breast carcinoma cell lines. We also investigated the effect of this newly synthesized CCH on the expression levels of TNF α, COX 2 and p53 in HepG2 and MCF7 cell lines.

## Materials and Methods

*Cell lines*

Liver cancer cell line HepG2 and breast cancer cell line MCF7 were purchased from Egyptian Company for Production of Vaccines, Sera, and Drugs, (VacSera). Cells were cultivated in high glucose Dulbecco’s modified Eagle’s medium (DMEM), augmented with 100 U/ml penicillin (both from Thermo Fisher Scientific, Inc., Waltham, MA, USA), 100 μg/ml strep¬tomycin (Sigma Aldrich), 2 mM L glutamine and 10% fetal bovine serum (both Thermo Fisher Scientific, Inc.) in a humidified 5% CO_2_ incubator at 37^o^C. Both cell lines were allowed to grow in plastic tissue culture T-flasks25 cm^2^ (Nunc) at 36^o^C and 5% CO_2_ (Kloskowski et al., 2010).

*Chemistry*

Ciprofloxacin chalcone hybrid (CCH) was prepared as previously reported (Abdel-Aziz et al., 2013). The synthetic pathway is through alkylation of ciprofloxacin with acylated chalcone derivative 1 in acetonitrile using triethylamine as a base (Scheme 1). The final product (CCH) was identified by 1H-NMR, 13C-NMR and mass spectrometry (Abdel-Aziz et al., 2013).

*Cell viability assay*

Assay was accomplished using MTT [3-(4,5-dimethylthiazol-2-yl)-2,5-diphenyltetrazolium bromide]. The stock solutions used in this research were diverse concentrations of CCH (in H2O). HepG2 and MCF7 cells in expanding phase were washed three times with phosphate buffered saline (PBS) then detached with 0.25% trypsin followed by centrifugation. The cells were re-suspended in RPMI- medium and were implanted (5×10^3^/well) into 96-well plate. The cells were incubated in a humidified atmosphere of 5% CO_2_ for minimum six hours, followed by the addition of CCH at final concentrations 2,000, 1,000, 500, 250, 125, 60, 30 and 15 μM and incubated for 24 h and 48 h at 37°C. After incubation, 10 μl MTT solution (1 mg/ml) was added to each well, followed by further incubation for 4 h. After incubation, the cell culture was evacuated by aspiration and 100 μl dimethyl sulfoxide (DMSO) was included in each well to resolve the formazan crystals (Zandi et al., 2017).

The cytotoxicity of CCH against cancer cell lines was set up on the principle of a comparison of the viability of the cells in the various concentrations of CCH to lifetime of cells derived from control and calculated from the formula:

X = LC/LK × 100%

Where X = cytotoxicity of CCH, LC = the average number of cells in the test sample, LK = the average number of cells in control (Kloskowski et al., 2010).

*Cell Cycle Analysis*

Liver cancer cell line HepG2 and breast cancer cell line MCF7 cells were seeded at a density of 6x10^5^ in 100-mm culture dishes and developed to 50%confluence. Thus, the cells were cultured in medium free of serum for 24 h then MCF7 and HepG2 cells treated with IC_50_, i.e., 54ug/ml and 22ug/ml of CCH respectively for 24–48 h in complete medium. The cells were gathered by trypsinization, subjected to centrifugation at 2000 rpm for 5 min, washed in PBS, and suspended in cold 70% ethanol.(Aranha et al., 2000). The cells were then analyzed by flow cytometry on FACStar Plus (Becton Dickinson, San Francisco, CA) after propidiumiodide staining. Cell termination was determined by lysing cells in a hypotonic solution containing 0.1% sodium citrate, 0.1% Triton X-100 and50 mg ml71 propidium iodide after washing the cells with PBS and Trypsin-EDTA solution two times. Analyzing the labelled nuclei was performed on a FACSCalibur fluorescence-activated cell sorter (FACS) using CELLQuest software (from Becton Dickinson). Apoptotic cells percentage was determined by measuring the portion of nuclei that contained a sub-diploid DNA content. Ten thousand events were collected for each sample analyzed (Herold et al., 2002b).

*Annexin V assay*

Apoptosis was assessed using Annexin V-FITC Apoptosis Detection Kit, according to the manufacturer’s protocol (Sigma-Aldrich, Germany). Annexin V-FITC kit permits fluorescent detection of annexin V bound to apoptotic cells and quantitative determination by flow cytometry. The Phosphatidylserine sites on the membrane surface was labeled by AnnexinV-FITC kit that uses annexin V conjugated with dye fluorescein isothiocyante (FITC). The kit includes propidium iodide (PI) to label the cellular DNA in necrotic cells wherever the cell membrane has been entirely compromised. This combination permits the differentiation among early apoptotic cells (annexin V positive, PI negative), late apoptosis (annexin V positive, PI positive), necrotic cells (annexin V negative, PI positive), and viable cells (annexin V negative, PI negative) The assay was done for both untreated and treated cells with the IC_50_ of the target drug for 24 hr (Dai et al., 2015).

*Protein Extraction and Western Blot Analysis*

HepG2 and MCF7cells were cultivated in complete medium and permitted totie up for 24 h, followed by the addition of MCF7 and HepG2 cells together with 54 μg/ml and 22ug/ml of CCH. Cells were incubated for 24 hours. Control cells were kept up in regular medium. Cells were collected by scratching the cells from culture dishes with a scraper and collected by centrifugation. Cells were resuspended in 125 mM Tris-HCl buffer, sonicated with 10–20% yield, and lysed using an equal volume of 8% SDS to make a final concentration of 4% SDS in the sample. Cell extracts were left to boil for 10 min, cooled by ice, and allowed to centrifuge at 2000 rpm for 5 min then the supernatant is collected. Quantification of sample protein content took place using the BCA protein assay kit (Pierce, Rockford, IL). Equal amounts of protein (20-30 µg of total protein) were exposed to 14% SDS-PAGE and transferred by electrophoresis to a nitrocellulose membrane (Schleicher and Schuell, Keene, NH). Membranes were blocked with 10% dry milk before incubation with antibodies to COX2 (1:1000 dilution; Abcam, Co), p53 (1:500 dilution; Abcam, Co), TNFα (1:1,000 dilution; Abcam, Co), and beta-actin (1:2,000 dilution; Abcam, Co), membranes were washed by TBST (Tris buffered saline, Tween 20), left to incubate with secondary antibodies and conjugated with peroxidase.To detect reactive bands, ECL chemiluminescence reagent was used (Amersham Pharmacia Biotech, Freiburg, Germany).Thus intensities of bands were analyzed using densitometry. Normalization was achieved to b- actin. Chemiluminescent detection system was used to detect the signal (Pierce)(Aranha et al., 2003).

*Reverse transcriptase-polymerase chain reaction (RT-PCR)*

To identify the underlying mechanism, especially in early phase, IC50 dose was applied with the Hep G2 and MFC7 cells with a short time period incubation. The RT-PCR was done to know the changes of cox2, p53 and TNFα at mRNA level. B-actin’s basal gene expression was set as control in untreated cells and analyzed with various levels of CCH after 24 h incubation. Total RNA from the cells was extracted utilizing Trizol reagent (Sangon, Shanghai, China) according to the Manufacturer’s protocol. The M-MLV reverse transcriptase (Rnase H-) model used three micrograms of total RNA for reverse transcription in a total quantity of 20 μl. (LifeFeng biological technology corporation, Shanghai, China). Aliquots of 2 μl cDNA were then amplified using the 2xTaq PCR kit to a total volume of 25 μl (LifeFeng biological technology corporation, Shanghai, China) following the manufacturer’s suggested circumstances. Cycling conditions: 94°C for 5 min, followed by 30 cycles of 94°C for 30 seconds, 53°C for 30 seconds and 72°C for 1 min, and a final extension of 72°C for 10 minutes. PCR products have been separated from the 1.5% agarose gel and viewed by DNA green staining. Tocan 360 gel imager was used (version 3.2.1 software)(Fu et al., 2013). Primers’ sequences (Fuccelli et al., 2015; Li et al., 2020; Oku et al., 2004)are listed in Table 1.

*Statistical analysis*

**Figure 1:**
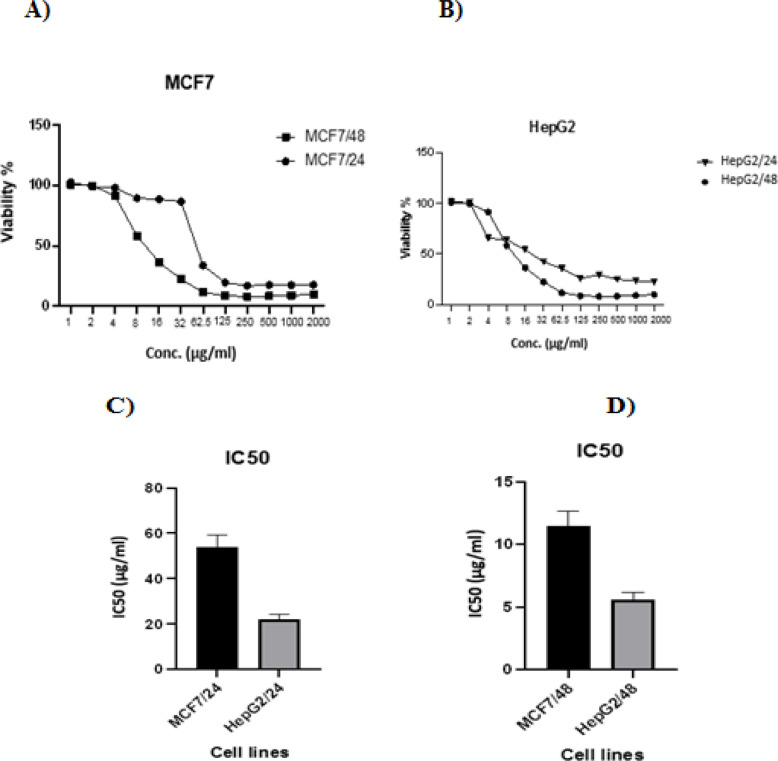
A, Viability percentage of MCF7 cells after 24 and 48 treatment with different concentrations of CCH; B, Viability percentage of HepG2 cells after 24 and 48 treatment with different concentrations of CCH; C, IC_50_ values of tested drug against after 24 hours in MCF7 and HepG2 cells; D, IC_50_ values of tested drug against after 48 hours in MCF7 and HepG2 cells

Results were collected from at least three separate experiments. Data were documented as mean± standard deviation. Differences were evaluated using Student’s t-test following a one-way study of variance (ANOVA), along with the use of GraphPad Prism5 statistical software (GraphPad, La Jolla, CA, USA) and Excel software (Microsoft, Redwood, WA, USA). Differences were said to be significant when the probability values (p) were less than 0.01 (Figure 1).

## Results

*Cell viability assay and IC50 for CCH in HepG2 and MCF7 cell lines*

In the case of HepG2 cell line, viability decreased significantly (p<0.001) with increasing concentration of the drug, but the half maximal inhibitory concentration, IC50 was in concentration of 22μg/mL and 5.6 μg/mL after 24 and 48 h of incubation, respectively. After 24 h incubation, the number of living cells was 22.35% with the highest CCH concentration. Only small number of living cells (4.75%) were noticed with CCH concentrations of 2,000 μg/mL and incubation time for 48 hours.

In the case of MCF7 cell line, viability decreased significantly (p<0.001) with increasing concentration of CCH. At low concentrations (1-30 μg/mL) cytotoxic effect of CCH was weak. At concentration 54 μg/mL, the number of cells decreased by about half compared to the control after 24 h of incubation. After 48 h of incubation cell viability was 15%. After 24 h incubation with the highest concentration of CCH, after 48 h the number of living cells was 17.87%. About 9.94% of living cells were observed with CCH concentrations of 2,000 μg/mL. CCH treatment showed important growth inhibition of HepG2 and MCF7 cell lines in dose-dependent manner compared to untreated cells (Figure 1).

Following 24 and 48 h of CCH treatment, the IC50 values were obtained based on the median-effect plot. After 24 h, the IC_50_ were 22±1.33 and 54±3.5 μg/ mL respectively for Hep G2 and MCF7 compared to doxorubicin (67.5±5.47 and 269.5±20.42). While the IC_50_ values for HepG2 and MCF7cell were 5.6±0.42 and 11.5±0.9μg / mL after 48 h CCH treatment compared to doxorubicin (15.3±1.33 and 70±5.5) (Table 2).

*Cell cycle analysis after CCH treatment in HepG2 and MCF7 Cells*

**Figure 2:**
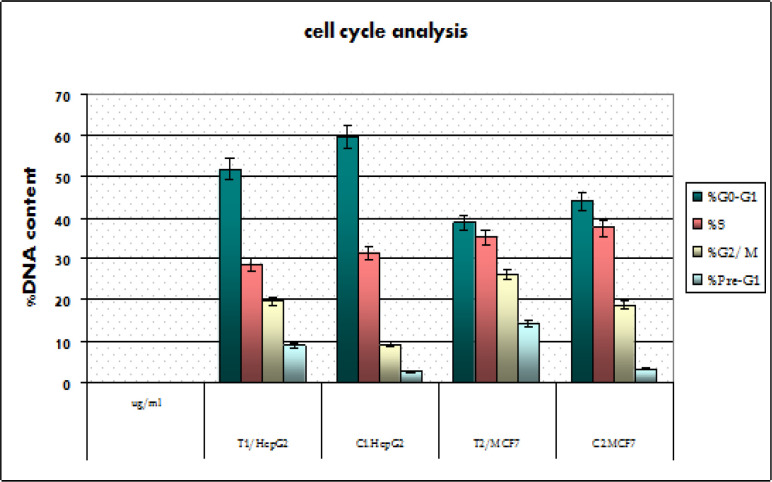
DNA Content in Different Phases of Cell Cycle after Treatment with CCH in IC_50 _Concentrations of 54ug/ml and 22ug/ml for MCF7 and HepG2 Cells respectively. Bar graph represents mean ± SEM from three independent experiments

**Figure 3:**
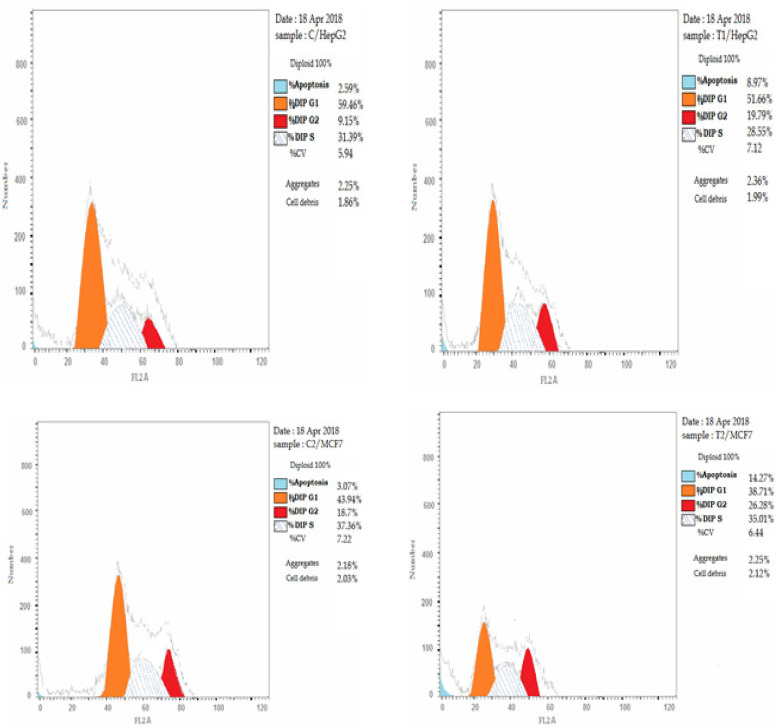
Cell Cycle Analysis, Effect of CCH on the Pases of Cell Cycle in Both MCF7 and HepG2 Cells

When MCF7 and HepG2 cell lines were treated with IC_50_ (54 ug/ml and 22ug/ml) of CCH respectively for 24h, we found a substantial amount of cells arrested during the G2/M phase. In untreated cells, 59.64% and 43.94 % of HepG2 and MCF 7 cells respectively were in G0-G1 phase, 31.39% and 37.36 % were in S phase, and 9.15 and 18.7% were in G2-M phase at 24hr. In CCH-treated cells, the number of cells in G0-G1 phase was reduced to 51.66 and 38.71%, the number of cells in S phase was reduced to 28.55 and 35.01% and the number of cells in G2/M phase was increased to 19.79% and 26.28% in HepG2 and MCF7 respectively after 24h of treatment. The outcomes of a typical experiment are summarized in (Figures 2 and 3).

DAPI nucleic staining was used to evaluate MCF7 and HepG2 cells treated with 54μg / ml and 22μg / ml of CCH for 24h showed significant pre-G1 apoptosis. The cells treated with CCH display nuclei fragmentation with this concentration. Significant pre G1 apoptosis was noted with IC_50_ dose of CCH after 24 hours (8.97±0.25%, p<0.05) for HepG2 cells and (14.27± 0.375%, p<0.05) for MCF7 cells compared to (2.59±0.25 and 3.07±0.25 %) of HepG2 and MCF7 in corresponding untreated control cells at 24h (Figures 2, 3).

*AnnexinV-FITC/propidium iodide analysis of apoptosis in both HepG2 and MCF7 cells*

**Figure 4:**
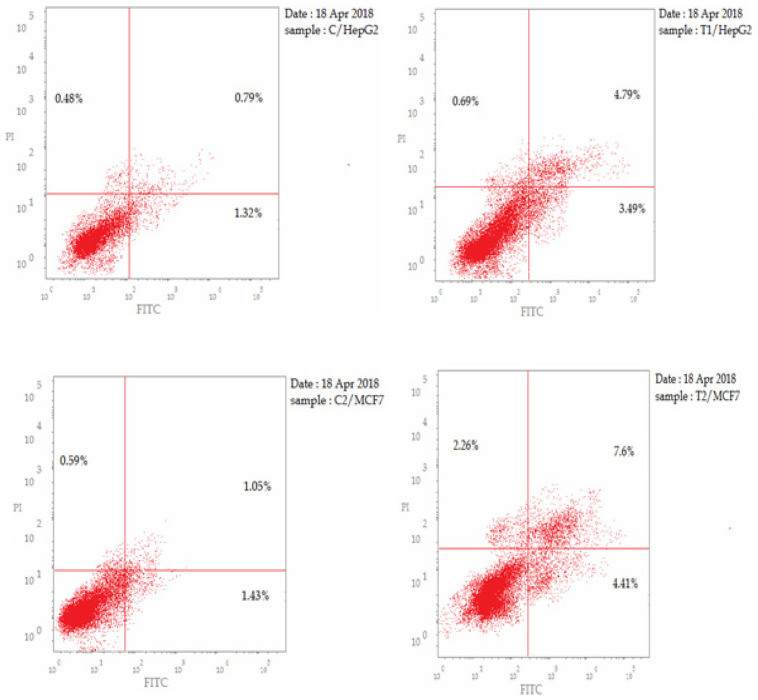
Effect of CCH on the Percentage of Annexin V-FITC-positive Staining in both HepG2 and MCF7 Cells. The experiments were done in triplicates. The four quadrants identified as: LL, viable; LR, early apoptotic; UR, late apoptotic; UL, necrotic

In this study, AnnexinV-FITC/PI dual staining assay was performed to evaluate the effect of CCH on both early and late apoptosis percentages in HepG2 and MCF7 cells. CCH treatment resulted in a significant increase (p<0.001) in the percentage of annexinV-FITC-positive apoptotic cells in HepG2 cells, including both the early and late apoptotic phases (LR; from 1.32% to 3.49%, and UR; from 0.76% to 4.79%), that represents about 4 fold total increase compared to untreated control. The same increase pattern was detected in MCF7 cells (LR; from 1.43% to 4.41%, and UR; from 1.05% to 7.6%), that represents about 5 fold total increase compared to untreated control (Table 3) and (Figure 4).

*Effects of CCH on protein expression of COX2, P53 and TNFα in MCF7 and HepG2 cells by western blotting*

**Figure 5:**
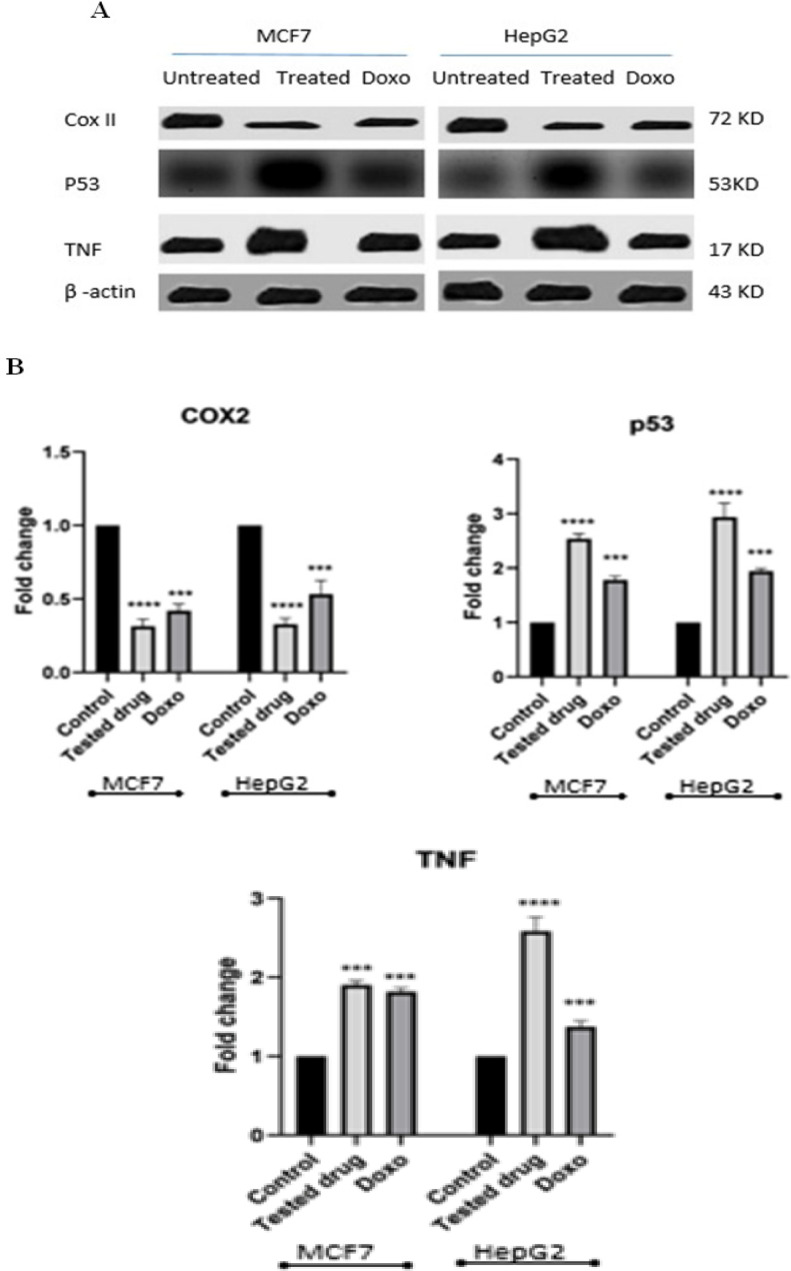
Expression of COXII, p53, and TNF Proteins in Untreated Cells, Cells which have been Treated for 24 hours with IC_50_ of CCH, and Cells Treated with Doxorubicin as a Positive Control. (A) Representative western blots for COXII, p53, and TNF in MCF7, and HepG2. β- actin was used as internal loading control, doxorubicin was used as positive control. (B) Relative protein expression in MCF7 and HepG2 after being treated for 24 hours with IC50 of CCH compared to untreated cells. β- actin protein expression was used to normalize expressed proteins. Doxorubicin was used as a positive control. Bars represent mean± SD. One way ANOVA test was used to analyze significant difference, where: ****p<0.0001, ***p<0.001, **p<0.01 compared to untreated cells

The protein expression levels of COX2, p53 and TNFαin both MCF7 and HepG2 cells treated without or with IC_50_ doses of CCH for 24 h was studied by western blot analysis, and compared to the effect of treating cell lines with doxorubicin as a positive control. P53 was significantly up-regulated in both treated cells compared to untreated controls (p<0.05). There was significantly lowered expression of COX2 in both treated cells compared to untreated controls (p<0.05). On the other hand, TNF-α showed significant higher expression in both treated cells compared to untreated cells (p>0.05)(Figure5).

*Effects of CCH on mRNA expression of COX2, P53 and TNFα in MCF7 and HepG2 cells by RT-PCR*

**Figure 6:**
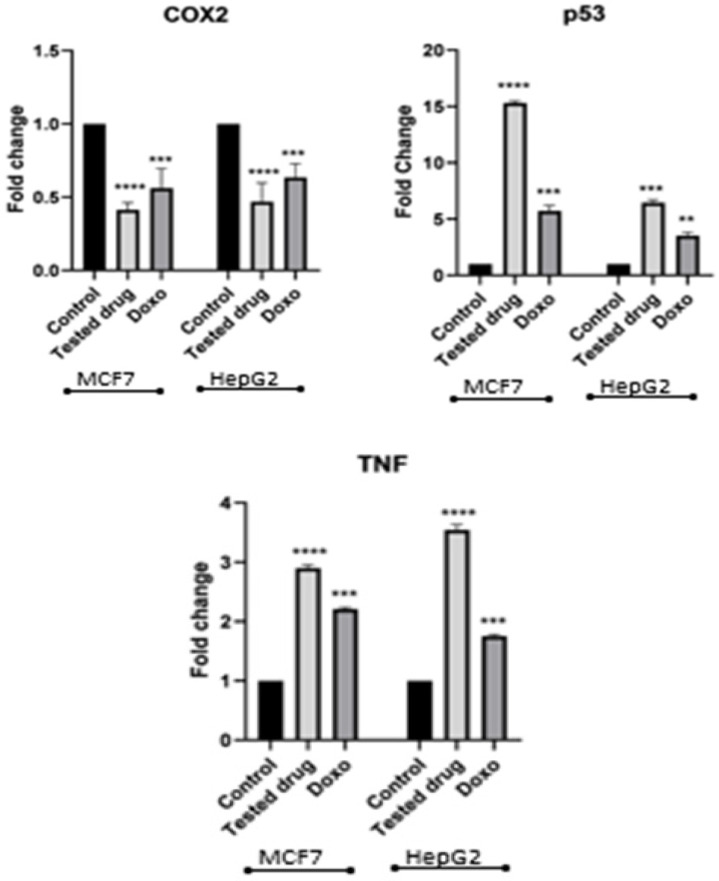
qRT-PCR Analysis of mRNA Expression Levels of COX_2_, p53 and TNF-α, in HepG2 and MCF7 Cells Treated with IC_50_ of CCH for 24 hours compared to Untreated Cells.Doxorubicin was used as positive control. Corresponding β- actin gene expression was used to normalize the tested gene expression. Bars show mean± SD. One-way ANOVA test was used to analyze significant difference, where; ****p<0.0001, ***p<0.001, and **p<0.01 compared to untreated cells

There was significantly lowered COX2 mRNA expression levels in both treated cells compared to untreated control (p<0.05), using doxorubicin as positive control. In contrast, p53 and TNFα were significantly up-regulated in both treated cells compared to untreated control (p<0.05)(Figure 6).

## Discussion

Despite huge revolution in cancer therapeutics, cancer is still a demanding approach (Nagura et al., 2013; Oba et al., 2020; Okabe et al., 2017; Otaka et al., 2013). Screening new natural or synthetic anti-cancer agents looking for better therapeutic options is still attractive for many researchers (Naseem et al., 2020; Othman et al., 2021). Fluoroquinolones are broad spectrum antibiotics which are active against multiple gram positive and gram negative bacteria, particularly by aiming bacterial DNA gyrase and topoisomerase IV (Blondeau, 2004). This mechanism of action seems to be helpful in treating certain kinds of malignant (Aranha et al., 2000; Yadav et al., 2015). Besides their antibacterial activity, FQs are also known to have several immunomodulatory impacts in different cell lines (Dalhoff, 2005). In addition, earlier studies highlighted the capacity of fluoroquinolones and their derivatives to cause apoptosis and cell cycle arrest in different cancer cell lines (Reuveni et al., 2010).

In the current study, we explored the impact of CCH on MCF7 and HepG2 cell lines on the basis of the information documenting the prospective cytotoxic impact of ciprofloxacin itself and because the molecular mechanism underlying the action of this medication is not brought to light fairly. MCF7 and HepG2 were selected because breast cancer and Hepatocellular carcinoma are widely spread in Egypt. We also investigated the effect of CCH on the expression levels of TNF α, COX 2 and p53 to elucidate the role of inflammation in cancer progression and how it impacts proliferation and apoptosis.

Firstly, we studied the effect of the synthesized CCH on cell viability, cell cycle distribution and apoptosis. It caused a time- and concentration-dependent decline in the viability of MCF7 and HepG2 cells. Similar findings have been noted with ciprofloxacin in previous studies, one of them on three cell lines of colorectal cancer (CC-531, SW-403, HT-29) (Aranha et al., 2003; Herold et al., 2002a). Another study on the cell line HTB9 of human bladder cancer (Aranha et al., 2000), and androgen independent prostate carcinoma PC3 cells (Aranha et al., 2003), colon carcinoma cell lines CC-531, SW-403 and HT-29 (Herold et al., 2002a) or pancreatic cancer cell lines MIA PaCa-2 and Panc-1(Yadav et al., 2015). Ciprofloxacin was used in concentrations of 400 μg/ml (above 1.0 mM). Significant decreases in cell viability and apoptosis induction were shown for levels of ciprofloxacin greater than 200 μg / ml (Beberok, Wrześniok, Minecka, et al., 2018). This effect may be explained by topoisomerase inhibitor anticancer activity which may happen through inhibition of mitochondrial DNA synthesis, which eventually induces mitochondrial injury, respiratory chain disorders, and depletion of ATP stores intracellular. Energy depletion promotes apoptosis because it can lead to the arrest of cells in the S- and/or G2/M phases (Fathy et al., 2017). 

Then, we further assessed whether cell cycle alterations could be related to the proven reduction in cell viability. We found that CCH caused G2/M phase cycle arrest in MCF7 and HEPG2 cells) suggesting a topoisomerase II inhibition mechanism (Fathy et al., 2017). Similarly, ciprofloxacin caused cells to be arrested at the G2/M checkpoint in human non-small lung cancer cells (Kloskowski et al., 2012). In addition, ciprofloxacin was found to be capable of inducing S-phase arrest as well as fragmentation of DNA in human pancreatic cancer cells (Yadav et al., 2015). This difference in mechanistic action could be ascribed to the difference in cell type origin. It has also been shown that different molecular pathways in the same cell line can be activated by fluoroquinolone derivatives (Blau et al., 2007). Koziel et al also showed that ciprofloxacin inhibited Jurkat cell proliferation in G2/M stage which impaired mitotic spindle formation and caused aneuploidy (Koziel et al., 2010). 

The cancer-related inflammation causes the release of inflammatory cells and inflammatory mediators, including chemokines, cytokines and prostaglandins, which are involved in angiogenesis and carcinogenesis (Fathy and Nikaido, 2013, 2018; Landskron et al., 2014). Among these cytokines, tumor necrosis factor-alpha (TNF-α) is a vital multifunctional cytokine (Eldafashi et al., 2021), which has an important role in regulation of cell survival, apoptosis, inflammation and immune reaction (Crusz and Balkwill, 2015). Cyclooxygenase-2 (COX-2), has a known role in chronic inflammation-related carcinogenesis. Over-expression of COX-2 has been noticed in various cancers (Yu et al., 2016). p53 protein induces cell-cycle arrest in the G1 phase and promotes DNA repair genes. If a cell with damaged DNA cannot be repaired, the cell is guided by p53 to experience apoptosis (Lukin et al., 2015). A few studies have identified complex relation between p53 and COX-2, whereby p53 may either up- or down-regulate COX-2, which conversely controls p53 transcriptional activity (Choi et al., 2005; de Moraes et al., 2007; Duarte et al., 2009).

In the current study, CCH also induced apoptosis in HepG2 and MCF7 cells through up-regulation of p53. Apoptotic cell death is one of the suggested anticancer therapeutic mechanisms of ciprofloxacin (Moustafa Fathy et al., 2019). This could be attributed to Bax up-regulation, modifying the Bax/Bcl-2 ratio favoring apoptosis (Zandi et al., 2017). Beberok et al have proved the cytotoxic impacts of ciprofloxacin on MDA-MB-231 breast cancer cells (Beberok et al., 2018). The researchers showed that ciprofloxacin in MDA-MB-231 cells at concentrations of 0.83 and 0.14 μmol / ml caused a 50 percent reduction in cell viability after 24 and 48 h incubation respectively. Cytotoxic impact of ciprofloxacin was independent of incubation period and antibiotic concentration in the case of hepatocellular carcinoma lineHepG2 (Kloskowski et al., 2011). Similar outcomes were achieved by Herold et al, where the mitochondrial apoptosis pathway mediated the cytotoxic response of human colorectal carcinoma cells to ciprofloxacin therapy (Herold et al., 2002a) and by Beberok et al, who identifyed that after 24 h treatment with ciprofloxacin, the percentage of late apoptotic cells rose to a peak (Beberok et al., 2018). One of the main reactions of drug-induced DNA damage is the expression of p53 that contributes to apoptosis induction through the mitochondrial intrinsic pathway. This may explain in part the proapoptotic effect of CCH (Aubrey et al., 2018).

In the current research, CCH caused G2/M cell cycle arrest in MCF7 and HepG2 cells (Evan and Vousden, 2001). Anticancer therapies can be used to interrupt the cancer cell cycle. It has been demonstrated by Kloskowski et al., (2011) that ciprofloxacin induces cell cycle arrest at the G2/M checkpoint in human non-small lung cancer cells. While reduced concentrations of ciprofloxacin caused S-phase cell cycle arrest in MDA-MB-231 cells (Beberok et al., 2018). Tumor tissues are heterogeneous thus depending on the type and origin of the cell, distinct molecular mechanisms of drug action may dominate (Noto et al., 2013; Wang et al., 2017). In addition, distinct molecular pathways in the same cell line can be triggered by separate fluoroquinolone derivatives (Beberok et al., 2017). Chemotherapy agents can behave in two ways: inhibit (cytostatic) cell growth or kill (cytotoxic) cells (Drlica, 1999). This anti-proliferative and pro-apoptotic activity of CCH in tumor cell lines could be mediated by S-G2/M cell cycle arrest. Bax translocation to mitochondrial membrane in some cancer cells leads to a rise in the Bax/Bcl-2 proportion (Aranha et al., 2003). 

The current results showed significant higher expression levels of TNF-α in both cell lines after treatment with CCH. TNF-α has various biological effects in different tissues ranging from inflammatory and cytotoxic impact to tissue remodeling and proliferation (Fathy et al., 2019; Fathy et al., 2020; Fathy et al., 2020). Some cancer cells are especially susceptible to TNF-α’s cytotoxic impact. This paradox is due to two distinct and separate TNF-α-mediated pathways in cells. One way contributes to apoptosis and the other leads to NF-kB activation of protective antiapoptotic genes. Which path is activated relies on that specific cell’s TNF-a threshold. Lower or normal concentrations of TNF-α typically stimulate the NF-kB protective pathway to prevent the cell from unnecessary apoptosis where greater or toxic concentrations cause apoptosis (Balkwill, 2006). TNF-α provokes a number of signal transmission processes, which lead to either apoptosis or proliferation according on TNF-α threshold of a cell. Most cells do not go through apoptosis when exposed to low levels of TNF-α (Muenchen et al., 2000). This is likely a protective mechanism by the cell based on TNF-α induction of anti-apoptotic genes. Apoptosis is induced by high levels of TNF-αbinding to its receptor, TNF-RI, and activating members of the caspase family of proteases (Soares et al., 1998). 

On the other hand, the current results showed significant lower expression levels of COX-2 after CCH treatment in both cell lines. Previous studies suggested that COX-2 may induce tumorgenesis by its inhibitory impacts on p53-induced apoptosis, and anti-tumorigenic effects of COX-2 inhibitors may be due to the potentiation of p53-induced apoptosis under genotoxic stresses (Choi et al., 2005). COX-2 is regarded as a powerful oxidizer and neighboring cellular substrates can be oxidized by the enzyme, resulting in DNA damage. Increased COX-2 concentrations can also lead to arachidonic acid metabolites depletion which can lead to decreased cell apoptosis (Yang et al., 2010). The oxidation stress is known to stimulate the activation of the TP53 gene and therefore the production of the protein p53 tumor suppressor, to avoid further damage to DNA (Vane et al., 1998). Previous research showed that COX-2 is a downstream p53 target protein, the expression of which is caused by different genotoxic stresses. Also, COX-2 inhibits apoptosis caused by p53- or DNA damage. These data proposed that COX-2 could stimulate tumorigenesis by inhibiting ordinary p53 function (Choi et al., 2005).

In conclusion, the current study, we investigated the anti-proliferative and pro-apoptotic effects of the synthesized 3,4,5 tri-methoxy ciprofloxacin chalcone hybrid (CCH) (Ar = 3,4,5-tri-OCH3-C6H2),on HepG2 and MCF7 cell lines and its effect on protein and mRNA expression levels of P53, COX-2 and TNF-α. CCH showed an obvious anti-proliferative activity as it caused a concentration and time-dependent reduction in the viability of human HepG2 and MCF7 cells. It also caused pre-G1 apoptosis and cell cycle arrest at G2/M stage. It also caused up-regulation of P53 mRNA and protein expression levels which may be a basic regulatory element for its apoptosis inducing effect. Higher mRNA and protein expression of TNF-α may participate in its apoptotic potential. In addition, CCH showed possible anti-inflammatory potential manifested by down-regulation of COX2 mRNA and protein expression levels, which may contribute partially to its anti-proliferative activity. Together, these findings provide a powerful experimental evidence for a possible anti-cancer therapeutic potential of CCH in HepG2 and MCF7 cells, giving a new insight into the therapeutic characteristics of this synthesized ciprofloxacin chalcone hybrid. Further research is needed to confirm these observations and to suggest detailed mechanistic interpretation for such effects. We believe this is significant because persistent chemoresistance of conventional chemotherapy is still a major obstacle in cancer therapeutics, thus recognition of novel anti-cancer agents is an urging continuous need.

**Table 1:** Primers Used in Real Time qPCR

Primers	Sequence of primers
COXII	Forward 5′-TGAAACCCACTCCAAACACAG-3′
	Reverse 5′-TCATCAGGCACAGGAGGAAG-3′
P53	Forward 5'-CACAGCGTGGTGGTACCTTATGAG-3'
	Reverse5'-TGGTAAGGATAGGTCGGCGGTTC-3'
TNF	Forward 5'-GAGCACTGAAAG CATGATCCG-3
	Reverse 5'-AAAGTAGACCTGCCCAGACTCGG-3'
β- actin	Forward 5'-GTGCTATGTTGCTCTAGACTTCG-3'
	Reverse 5'- ATGCCACAGGATTCCATACC-3'

**Table 2:** *In vitro *Anti-Proliferative Activity and IC_50 _of CCH after 24 and 48 hours in both HepG2 and MCF7 Cell Lines

	IC_50_ (μg/mL)#			
Cell line	HepG2		MCF7	
Incubation time	24 hrs	48 hrs	24 hrs	48 hrs
CCH	22±1.33	5.6±0.42	54±3.5	11.5±0.9
Doxorubicin	67.5±5.47	15.3±1.33	269.5±20.42	70±5.5

# IC_50_ values are the mean ± SD of three separate experiments

**Table 3:** Distribution of Apoptotic Cells in the Annexin V-FITC Experiment in Both in Both HepG2 and MCF7 Cell Lines

	Apoptosis			Necrosis
	Early Apoptosis (Lower Right %)	Late Apoptosis (Upper Right %)	Total (L.R % ‏ U.R %)	
CCH/HepG2	3.49	4.79	8.97	0.69
HepG2	1.32	0.79	2.59	0.48
CCH/MCF7	4.41	7.60	14.27	2.26
MCF7	1.43	1.05	3.07	0.59

## Author Contribution Statement

Marwa A. Eisa: Collected the data, verified analysis tools, performed analysis, performed statistical analysis and interpretation of data, contributed in paper writing and drafting. Maiiada H. Nazmy: Conceived the presented idea, proposed the experimental design, verified the analytical methods, supervised analytical performance, contributed in data interpretation, contributed in writing, drafting, revision and submission of the paper. Moustafa Fathy: Conceived the presented idea, proposed the experimental design, verified the analytical methods. Gamal El-Din A. A. Abu-Rahma: Chemical synthsis of 3,4,5 tri-methoxy ciprofloxacin chalcone hybrid. Mohamed Abdel-Aziz: Chemical synthsis of 3,4,5 tri-methoxy ciprofloxacin chalcone hybrid
